# Co-Inhibition of GLUT-1 Expression and the PI3K/Akt Signaling Pathway to Enhance the Radiosensitivity of Laryngeal Carcinoma Xenografts *In Vivo*

**DOI:** 10.1371/journal.pone.0143306

**Published:** 2015-11-24

**Authors:** Xing-Mei Luo, Bin Xu, Min-Li Zhou, Yang-Yang Bao, Shui-Hong Zhou, Jun Fan, Zhong-Jie Lu

**Affiliations:** 1 Department of Otolaryngology, the First Affiliated Hospital, College of Medicine, Zhejiang University, Hangzhou, Zhejiang Province, 310003, China; 2 State Key Laboratory for Diagnosis and Treatment of Infectious Diseases, The First Affiliated Hospital, College of Medicine, Zhejiang University, Zhejiang Province, 310003, China; 3 Department of Radiotherapy, the First Affiliated Hospital, College of Medicine, Zhejiang University, Hangzhou, Zhejiang Province, 310003, China; University of Navarra, SPAIN

## Abstract

In the present study, we investigated the role of GLUT-1 and PI3K/Akt signaling in radioresistance of laryngeal carcinoma xenografts. Volume, weight, radiosensitization, and the rate of inhibition of tumor growth in the xenografts were evaluated in different groups. Apoptosis was evaluated by TUNEL assay. In addition, mRNA and protein levels of GLUT-1, p-Akt, and PI3K in the xenografts were measured. Treatment with LY294002, wortmannin, wortmannin plus GLUT-1 AS-ODN, and LY294002 plus GLUT-1 AS-ODN after X-ray irradiation significantly reduced the size and weight of the tumors, rate of tumor growth, and apoptosis in tumors compared to that observed in the 10-Gy group (p<0.05). In addition, mRNA and protein expression of GLUT-1, p-Akt, and PI3K was downregulated. The E/O values of LY294002, LY294002 plus GLUT-1 AS-ODN, wortmannin, and wortmannin plus GLUT-1 AS-ODN were 2.7, 1.1, 1.8, and 1.8, respectively. Taken together, these data indicate that GLUT-1 AS-ODN as well as the inhibitors of PI3K/Akt signaling may act as radiosensitizers of laryngeal carcinoma *in vivo*.

## Introduction

Radiotherapy plays an important role in the treatment of early-stage laryngeal carcinoma and laryngeal preservation [1]. However, radioresistance always ultimately leads to treatment failure [2–4]. Although there are various methods to improve the effects of radiotherapy, including hyperfractionation[5], concurrent chemoradiotherapy [6], and some radiosensitizers [7], the results of these treatments are unsatisfactory. Thus, there is a continuing need for new strategies to improve the radiosensitivity of laryngeal carcinoma and decrease the side effects of radiotherapy.

However, the mechanism(s) of radioresistance in laryngeal carcinoma remain(s) unclear. The mechanism may result from interactions of multiple factors, such as hypoxia, intrinsic radioresistance, including repopulation, tumor-cell proliferation, and DNA damage repair[8]. Glucose transporter-1 (GLUT-1) has been demonstrated to be an important hypoxic marker in malignant tumors, including laryngeal carcinoma[9–11]. Previous studies, including ours, have revealed that high expression of GLUT-1 may be associated with chemoradioresistance in some cancers[12–14]. Several reports have found that targeted inhibition of GLUT-1 expression may suppress the growth and proliferation of cancer cells[14–17]. To our knowledge, we were the first to report a relationship between GLUT-1 expression and radioresistance and revealed that inhibition of GLUT-1 expression by antisense oligodeoxynucleotides (AS-ODN) may improve the radiosensitivity of laryngeal carcinoma, *in vitro* and *in vivo*[1].

As mentioned above, radioresistance may be the result of multiple factors. Thus, high GLUT-1 expression is likely not the only factor in resistance to radiotherapy in laryngeal carcinoma. Some studies have revealed that the phosphatidylinositol 3-kinase/protein kinase B (PI3K/Akt) pathway plays a regulatory role in GLUT-1 localization [18, 19]. Activation of the PI3K/Akt pathway may be associated with cancer radioresistance[20–22]. We reported previously that resistance or insensitivity of Hep-2 cells to cisplatin may be associated with GLUT-1 expression and the PI3K/Akt pathway [1]. We also found that apigenin may enhance the sensitivity of laryngeal carcinoma cells to cisplatin via inhibition of GLUT-1 and p-Akt expression. However, no data on enhancing radiosensitivity by combined inhibition of PI3K/Akt and GLUT-1 expression in carcinomas have been reported to date.

In this study, we detected the expression of GLUT-1 and molecules of the PI3K/Akt signal pathway in a xenograft model of human laryngeal carcinoma constructed in nude mice. We further investigated whether GLUT-1 expression and the PI3K/Akt signaling pathway played a role in radioresistance in xenografts of laryngeal carcinoma and whether targeted inhibition of GLUT-1 expression and the PI3K/Akt pathway can enhance the radiosensitivity of laryngeal carcinoma *in vivo*.

## Materials and Methods

### Cell culture

The laryngeal carcinoma Hep-2 cell line was purchased from the Cell Research Institute of Chinese Academy of Sciences (Shanghai, China). Hep-2 cells were cultured in Dulbecco’s modified Eagle’s medium (DMEM; GIBCO-BRL, Gaithersburg, MD) containing 10% heat-inactivated fetal bovine serum (FBS, Hyclone, Logan, UT), 2 mM L-glutamine, 100 U/mL penicillin, and 100 μg/mL streptomycin at 37°C in a 5% CO2 atmosphere. Cells were trypsinized and harvested after reaching 80–90% confluency[1].

### Antisense oligonucleotides to GLUT-1 and Ly294002 and wortmannin preparation

Antisense oligonucleotides to GLUT-1 were prepared according to our previous report1[17]. Ly294002 and wortmannin (specific inhibitor of PI3K/Akt signaling pathway) were purchased from Sigma (USA).

### Nude mouse model of laryngeal carcinoma

This experiment was conducted in accordance with the guidelines of the First Affiliated Hospital, College of Medicine, Zhejiang University. All animal experiments were conducted in accordance with the Guidelines for the Care and Use of Laboratory Animals and were approved by the Animal Ethics Review Committees of the First Affiliated Hospital, College of Medicine, Zhejiang University.

Approximately 2×106 Hep-2 cells were inoculated subcutaneously into the flanks of 4-week-old male athymic nude mice (specific pathogen-free (SPF)-grade BALB/c, weight 18±2 g). Tumor sizes were tracked using electronic calipers each day. The xenograft was regarded as a ‘success’ when the tumor size reached 5 mm. Tumor volumes were calculated using the formula: ½ (width^2^ × length).

### Groups

When the tumors reached a volume of ~100 mm3, 33 tumor-bearing mice were divided randomly into 11 groups each of three nude mice: negative control, tumors injected with GLUT-1 AS-ODN alone, tumors injected with Ly294002 alone, tumors injected with wortmannin alone, tumors injected with Ly294002 and GLUT-1 AS-ODN, tumors injected with wortmannin and GLUT-1 AS-ODN, without irradiation, and irradiation groups: 10-Gy X-ray irradiation alone, tumors injected with Ly294002 combined with 10-Gy X-ray irradiation, tumors injected with wortmannin combined with 10-Gy X-ray irradiation, tumors injected with Ly294002 and GLUT-1 AS-ODN combined with 10-Gy X-ray irradiation, and tumors injected with wortmannin and GLUT-1 AS-ODN combined with 10-Gy X-ray irradiation.

### Drug administration conditions

GLUT-1 AS-ODNs were injected intratumorally, 100 μg per time per mouse, three times at intervals of 2 days. Ly294002 and wortmannin were injected intraperitoneally (ip), 2 mg or 0.12 mg per time per mouse, respectively, three times at intervals of 2 days.

### Irradiation conditions

X-ray radiation was from a 6-MV linear accelerator (Clinac 23EX, Varian Inc., Palo Alto, CA, USA) at a dose of 10 Gy. The source-skin distance was 100 cm, the radiation field was 35 × 35 cm, there was a single-energy 6 MV X-ray, and the dose-rate was 500 MU/min. Animal irradiation was performed under general anesthesia (50 mg/kg ip injection of pentobarbital sodium; Hengrui Pharmaceutical Co., Shanghai, China). The drugs were administered as above. On day 10 (4 days after final drug administration), 10-Gy X-ray irradiation was administered per mouse.

### Tumor parameters

A tumor growth curve was drawn according to tumor volumes at each observation time (1, 5, 10, 17, and 24 days after treatment). At 27 days (2 weeks after X-ray irradiation), mice were sacrificed and the tumor was excised and weighed, and tumor samples were snap-frozen in liquid nitrogen. The inhibition ratio (IR) was defined as 1—(tumor weight of test group / control group)[23].

### Calculating radiosensitization

Radiosensitization was determined by the expected value/observed value (E/O value), as reported by Xia et al.[24], for Ly294002, wortmannin, and GLUT-1 AS-ODN. When E/O was > 1.4, we defined this as radiosensitization. E/O = (T2×T3) / (T4×T1) where T1, T2, T3, and T4 are the average tumor weights in the control group, the treatment group without 10-Gy X-ray irradiation, 10-Gy X-ray irradiation alone, and treatment group combined with 10-Gy X-ray irradiation, respectively.

### Apoptosis of tumor samples detected by TUNEL

Terminal deoxynucleotidyltransferase-mediated dUTP digoxigenin nick-end-labeling (TUNEL) staining of tumor sections was performed using an *in situ* apoptosis detection kit (Roche, Shanghai, China) according to the manufacturer’s protocol. Briefly, paraffin wax-embedded sections of tumor samples of xenografts underwent dewaxing, antigen retrieval, endogenous peroxidase activity blocking, TUNEL reaction, DAB staining, counting, and photographing. Cells where the nuclei were brown or brown-yellow or where the cytoplasm included a few brown or brown-yellow granules were interpreted as positive. The sections were observed under ×200 magnification and the apoptosis rate (number of TUNEL-positive cells/number of all cells) was calculated in each section.

### GLUT-1 mRNA, p-Akt mRNA, and PI3K mRNA detection by real-time RT-PCR

Real-time RT-PCR was performed as described previously[1]. Briefly, 50-mg tumor samples were homogenized in TRIzol reagent (Invitrogen, Carlsbad, CA). Total RNA was extracted according to the manufacturer’s protocol. The concentration and quality of total RNA was measured by ultraviolet spectrophotometry: an optical density (OD) 260/280 ratio between 1.8 and 2.1 was deemed to be acceptably pure. Reverse transcription was performed according to the manufacturer’s protocol. Total RNA (l μg) and Moloney murine leukemia virus (MMLV) reverse transcriptase (Fermentas, Canada) were mixed in a 20-μL reaction volume consisting of 0.5 μg/μL of oligo(dT) primer, 1 μL of random primers (0.2 μg/μL), and 10 μL of DEPC-treated H2O. The reaction mix was first pre-denatured at 65°C for 10 min. After addition of 200 U MMLV reverse transcriptase, the samples were incubated at 42°C for 1 h and annealed at 70°C for 10 min. The cDNA was then used as a template for real-time fluorescent quantitative PCR using the fluorescent dye SYBR Green and the Eppendorf Realplex 4 real-time PCR system (Hamburg, Germany). The 20-μL reaction mix consisted of 10 μL of 2×SYBR Green, 1 μL of template, 1 μL of upstream and downstream specific primers, and 8 μL of deionized water. The reaction mix was pre-denatured at 95°C for 5 min, followed by 40 cycles at 95°C for 15 s, 59°C for 20 s, and 72°C for 20 s. Each primer sample was run in triplicate. Primers sequences were as follows: GAPDH (control), sense 5’-TGTTGCCATCAATGACCCCTT-3’, antisense 5’-CTCCACGACGTACTCAGCG-3’ (202 bp), GLUT-1, sense 5’-GTCAACACGGCCTTCACTG-3’, antisense 5’-GGTCATGAGTATGGCACAACC-3’ (111 bp), p-Akt, sense 5’-GCAGCACGTGTACGAGAAGA-3, antisense 5’-GGTGTCAGTCTCCGACGTG-3’ (67 bp), and PI3K sense 5’-GGGGATGATTTACGGCAAGATA-3’, antisense 5’-CACCACCTCAATAAGTCCCACA-3’ (144 bp). To distinguish between specific and non-specific products and primer dimers, a dissociation curve analysis was conducted immediately after amplification by continuous monitoring of the SYBR Green I fluorescence signal at temperatures between 60 and 95°C. For calculation of differential gene expression, the 2-ΔΔCt formula was used.

### GLUT-1, p-Akt, and PI3K Western blotting

Western blotting was performed as described previously [1]. GLUT-1, p-Akt, PI3K, and β-actin (control) protein in each group of tumor cells were assayed using a BAC protein quantitative kit (Wuhan Boster Biological Technology Co. Ltd., Wuhan China). Briefly, 80 μg of protein was subjected to 10% sodium dodecyl sulfate-polyacrylamide gel electrophoresis (SDS-PAGE) and transferred onto a nitrocellulose membrane (Millipore, Billerica, MA, USA). Skimmed milk (2%) was used as a blocking solution (room temperature, 1 h). The membrane was incubated with the primary antibody (GLUT-1, 1:1000, p-Akt, 1:1000, PI3K, 1:800, β-actin, 1:4000) at room temperature for 3 h, and with the secondary antibody (1:5000, donkey anti-rabbit; 1:2000, donkey anti-mouse) at room temperature for 1h. The proteins were then detected using an enhanced chemiluminescence system (Santa Cruz Biotechnology, Santa Cruz, CA, USA) and were exposed to X-ray film. Protein expression was analyzed semi-quantitatively using the Kodak Gel Logic Analysis System.

### Statistical Analysis

Statistical analyses were performed using the SPSS software (ver. 19.0 for Windows; SPSS Inc., Chicago, IL). A P-value < 0.05 was deemed to indicate statistical significance.

## Results

### Volume, weight, and the rate of tumor growth inhibition in the xenograft

**Fig 1:**
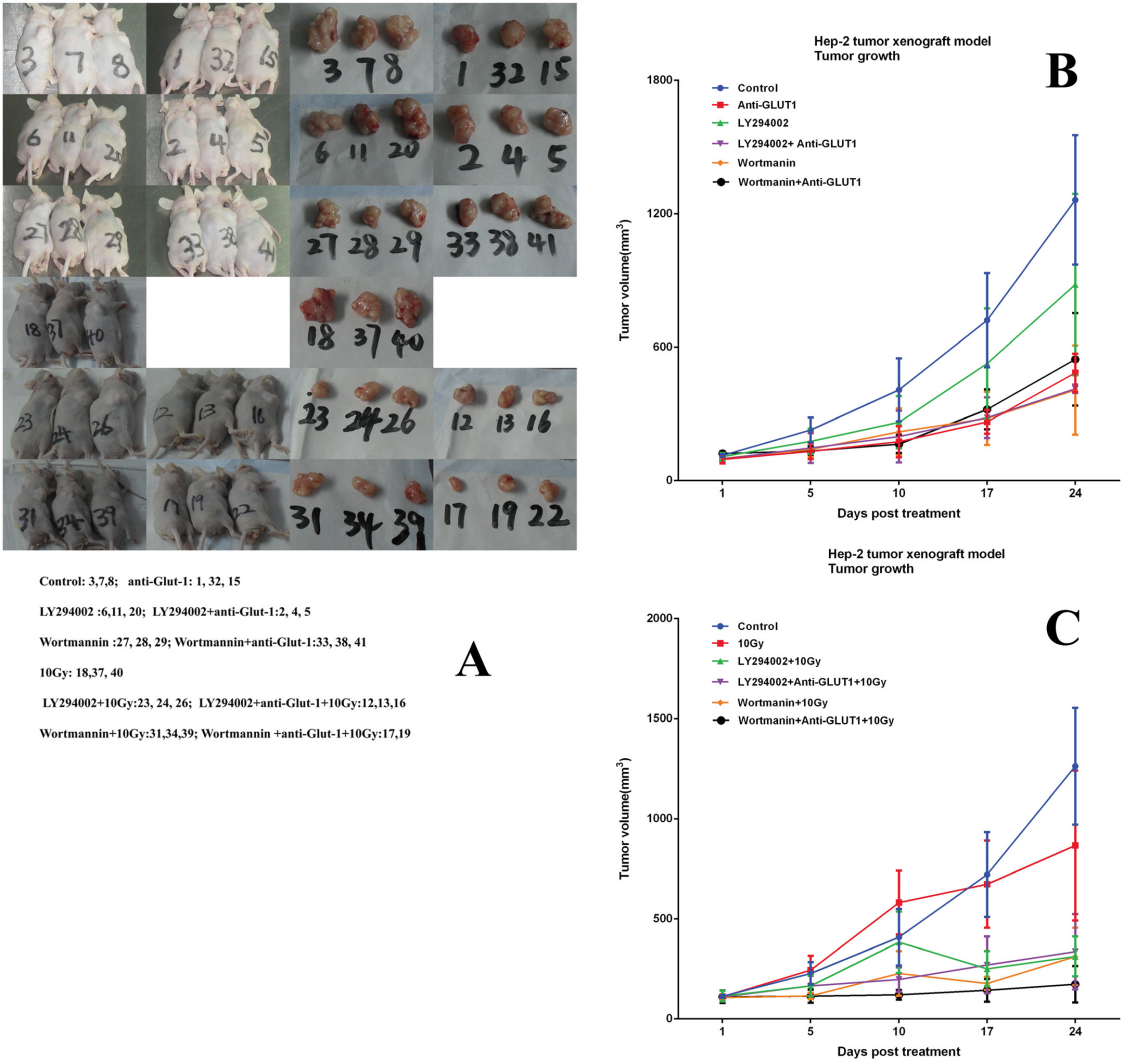
Effect of GLUT-1 AS-ODN, LY294002, wortmannin, 10Gy, LY294002 plus GLUT-1 AS-ODN, and Wortmannin plus GLUT-1 AS-ODN on volume, weight, and the rate of tumor growth inhibition in the xenograft. (A) mice and tumors. (B)before radiation. (C) after radiation.

The tumor formation rate was 100%. Without X-ray irradiation, GLUT-1 AS-ODN, wortmannin, and LY294002 plus GLUT-1 AS-ODN reduced the size of the tumors significantly, compared with the control after 17 days of treatment (p < 0.05, Fig 1). Wortmannin plus GLUT-1 AS-ODN reduced the size of the tumors significantly compared with control after 10 days treatment (p < 0.05). However, Ly294002 alone did not reduce the size of tumors significantly compared with the control (p > 0.05). The differences between GLUT-1 AS-ODN and LY294002 plus GLUT-1 AS-ODN, between GLUT-1 AS-ODN and wortmannin plus GLUT-1 AS-ODN, between wortmannin and wortmannin plus GLUT-1 AS-ODN, between LY294002 and LY294002 plus GLUT-1 AS-ODN on the sizes of tumors were not statistically significant (p > 0.05, Fig 1).

After X-ray irradiation, there was no statistically significant difference between the size of tumors in the 10-Gy group and the control group (p > 0.05). Ly294002 alone reduced the size of tumors significantly compared with the 10-Gy group after 17 days treatment (p < 0.05, Fig 1). Wortmannin, LY294002 plus GLUT-1 AS-ODN reduced the size of tumors significantly compared with the 10-Gy group after 10 days treatment (p < 0.05, Fig 1). Wortmannin plus GLUT-1 AS-ODN reduced the size of tumors significantly versus the 10-Gy group after 5 days of treatment (p < 0.05, Fig 1).

When the mice were sacrificed at 27 days after treatment, GLUT-1 AS-ODN, wortmannin, LY294002 plus GLUT-1 AS-ODN, and wortmannin plus GLUT-1 AS-ODN reduced the weight of tumors significantly compared with the 10-Gy group (p < 0.05, Fig 1). However, Ly294002 alone did not reduce the weight of tumors significantly compared with the 10-Gy group (p > 0.05). The differences between GLUT-1 AS-ODN and LY294002 plus GLUT-1 AS-ODN, between GLUT-1 AS-ODN and wortmannin plus GLUT-1 AS-ODN, between wortmannin and wortmannin plus GLUT-1 AS-ODN, and between LY294002 and LY294002 plus GLUT-1 AS-ODN in the weights of the tumors were not significant (p > 0.05, Fig 1). After irradiation, LY294002, wortmannin, LY294002 plus GLUT-1 AS-ODN, and wortmannin plus GLUT-1 AS-ODN reduced the weight of tumors significantly compared with the 10-Gy group (p < 0.05, Fig 1).

The rates of tumor growth inhibition in the xenograft of the GLUT-1 AS-ODN group, LY294002 group, LY294002 plus GLUT-1 AS-ODN group, 10-Gy group, LY294002 plus 10-Gy group, and LY294002 plus GLUT-1 AS-ODN plus 10-Gy group were 37.3%, 3.4%, 50.0%, 7.0%, 67.7%, and 57.8%, respectively. LY294002 plus GLUT-1 AS-ODN enhanced the effect of tumor growth inhibition significantly compared with GLUT-1 AS-ODN and LY294002 alone Without X-ray irradiation (p < 0.05).

After X-ray irradiation, LY294002 and LY294002 plus GLUT-1 AS-ODN enhanced the effect of tumor growth inhibition significantly, compared with 10 Gy (p < 0.05). The rates of tumor growth inhibition in the xenograft of the GLUT-1 AS-ODN group, wortmannin group, wortmannin plus GLUT-1 AS-ODN group, 10-Gy group, wortmannin plus 10-Gy group, and wortmannin plus GLUT-1 AS-ODN plus 10-Gy group were 37.3%, 53.4%, 43.5%, 7.0%, 68.1%, and 70.0%, respectively.

After X-ray irradiation, wortmannin plus GLUT-1 AS-ODN enhanced the effect of tumor growth inhibition significantly compared with 10 Gy and wortmannin (p < 0.05). The E/O values of LY294002, LY294002 plus GLUT-1 AS-ODN, wortmannin, and wortmannin plus GLUT-1 AS-ODN were 2.7, 1.1, 1.8, and 1.8, respectively.

### Effects of GLUT-1 AS-ODN, LY294002, and wortmannin on apoptosis in the xenograft

**Fig 2:**
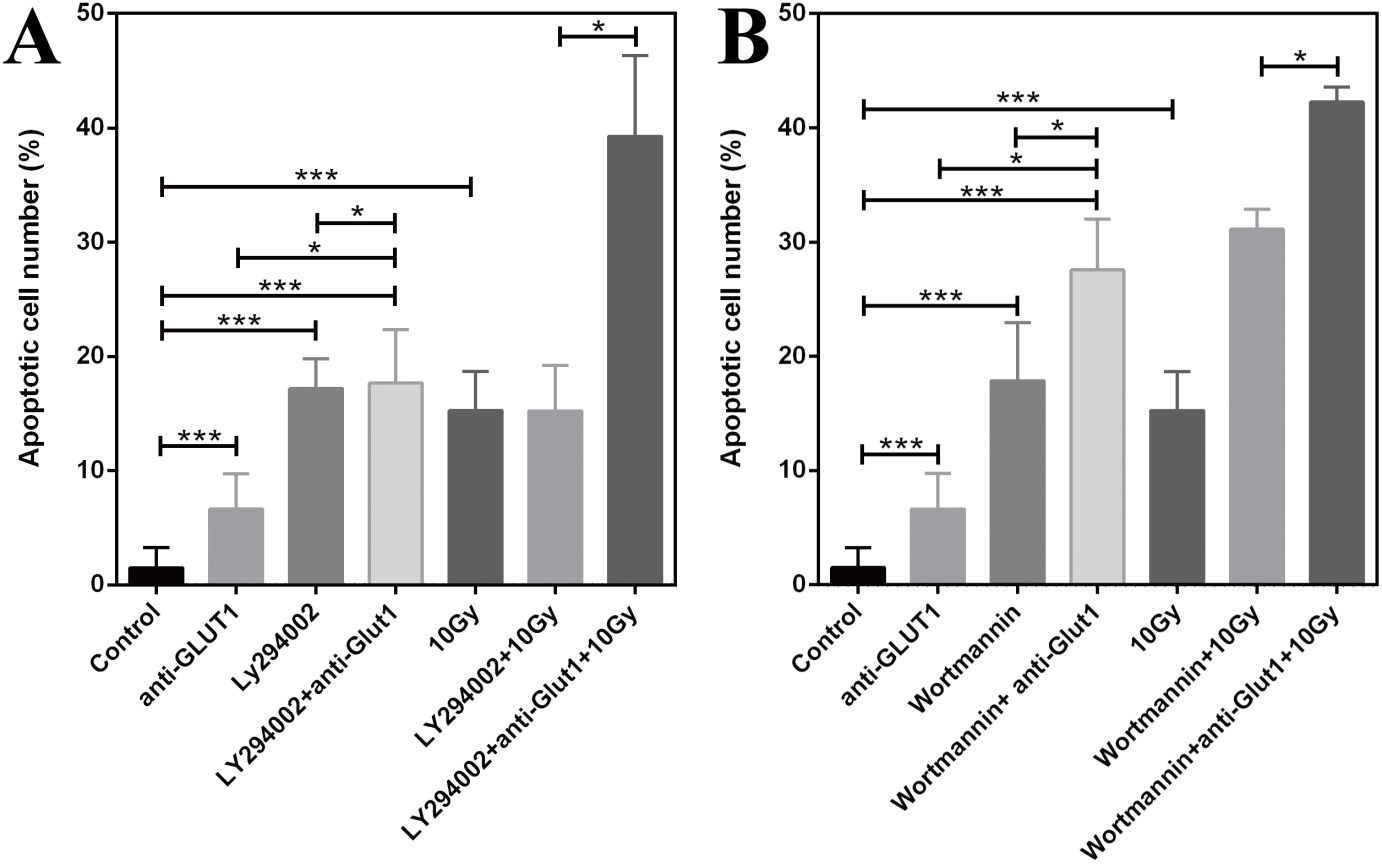
Effects on apoptosis in the xenograft. (A) GLUT-1 AS-ODN, LY294002. (B) GLUT-1 AS-ODN, wortmannin.

Without X-ray irradiation, the apoptotic rates with of GLUT-1 AS-ODN transfection, Ly294002 and wortmannin treatment, Ly294002 plus GLUT-1 AS-ODN, and wortmannin plus GLUT-1 AS-ODN were higher than in the control (p < 0.001, Fig 2). The apoptotic rate of Ly294002 plus GLUT-1 AS-ODN enhanced the effect of Ly294002 or GLUT-1 AS-ODN alone significantly on the apoptosis of tumor cells (p < 0.05, Fig 2). The apoptotic rate of wortmannin plus GLUT-1 AS-ODN enhanced the effect of wortmannin or GLUT-1 AS-ODN alone significantly on the apoptosis of tumor cells (p < 0.05, Fig 2).

After X-ray irradiation, the apoptotic rates in the 10-Gy X-ray irradiation group were higher than that in the control (p < 0.001, Fig 2). GLUT-1 AS-ODN may significantly enhance the apoptotic rate of Ly294002 or wortmannin on X-ray irradiation (p < 0.05, Fig 2).

### Effects of GLUT-1 AS-ODN, LY294002, and wortmannin on the expression of GLUT-1, p-Akt, and PI3K mRNA in the xenograft

**Fig 3:**
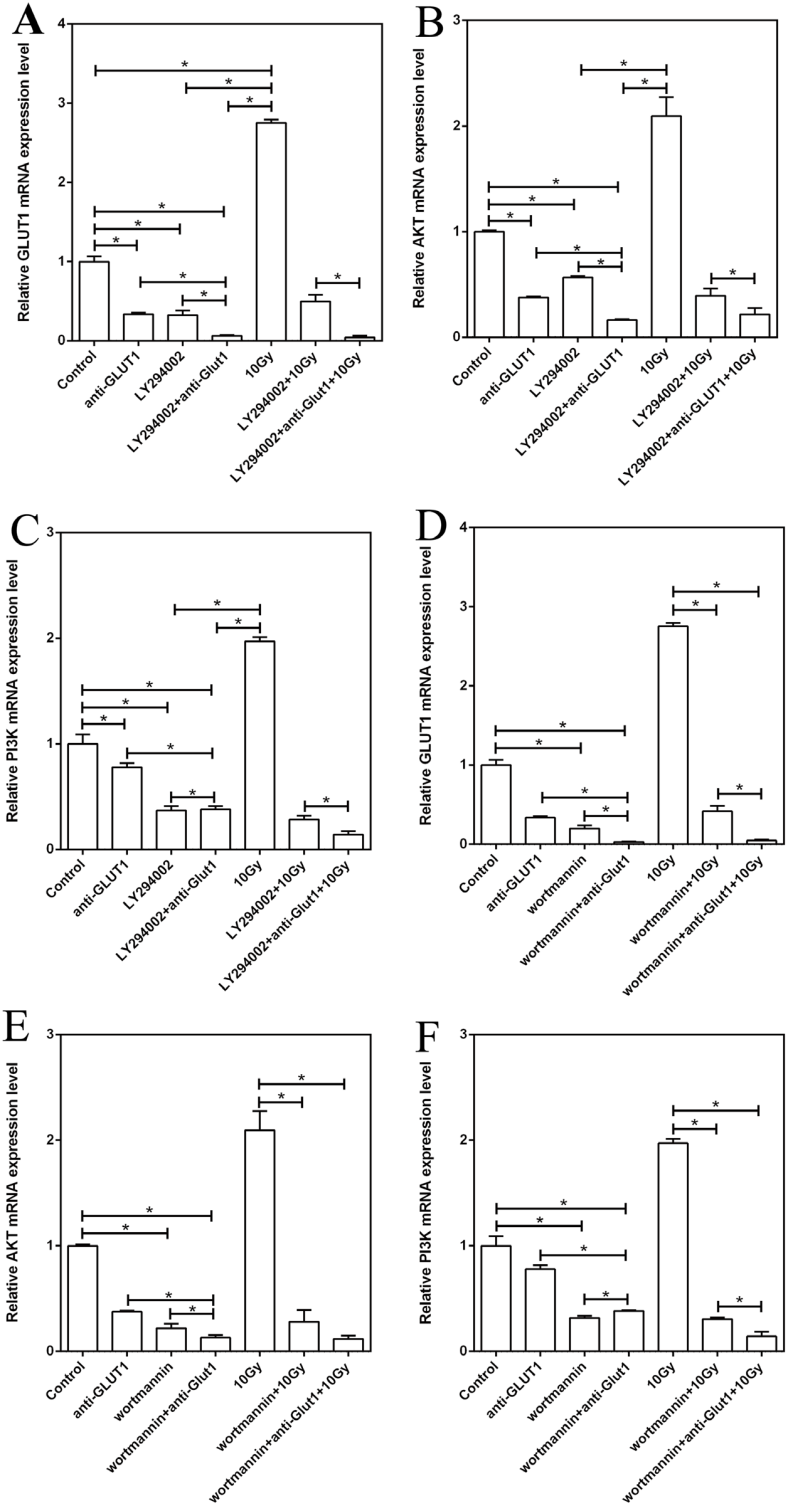
Effects of GLUT-1 AS-ODN, LY294002, and wortmannin on the expression of GLUT-1, p-Akt, and PI3K mRNA in the xenograft. (A) The effect of LY294002 on GLUT-1 mRNA expression. (B) The effect of LY294002 on p-Akt mRNA expression. (C) The effect of LY294002 on PI3K mRNA expression. (D) The effect of wortmannin on GLUT-1 mRNA expression. (E) The effect of wortmannin on p-Akt mRNA expression. F: The effect of wortmannin on PI3K mRNA expression.

Without X-ray irradiation, the expression of GLUT-1, p-Akt, and PI3K mRNA after transfection of GLUT-1 AS-ODN was lower than in the control group (p < 0.05, Fig 3). The expression of GLUT-1, p-Akt, and PI3K mRNA in the Ly294002 alone group and the Ly294002 plus GLUT-1 AS-ODN was lower than in the control group (p < 0.05, Fig 3). Ly294002 plus GLUT-1 AS-ODN enhanced the effect of inhibiting the expression of GLUT-1, p-Akt, and PI3K mRNA significantly versus Ly294002 or GLUT-1 AS-ODN alone (p < 0.05, Fig 3).

After X-ray irradiation, 10-Gy X-ray irradiation caused significantly higher expression of GLUT-1 mRNA versus the control group (p < 0.05). The expression of PI3K and p-Akt mRNA in the 10-Gy X-ray irradiation group was higher than the control group, albeit not significantly so. (p > 0.05). Ly294002 alone or Ly294002 plus GLUT-1 AS-ODN decreased the expression significantly of GLUT-1, PI3K, and p-Akt mRNA compared with the 10-Gy X-ray group (p < 0.05, Fig 3). Ly294002 plus GLUT-1 AS-ODN enhanced the effect of inhibition of expression significantly of GLUT-1, PI3K, and p-Akt mRNA versus Ly294002 alone after 10-Gy X-ray irradiation (p < 0.05, Fig 3).

Without X-ray irradiation, the expression of GLUT-1, p-Akt, and PI3K mRNA in the wortmannin alone group and the wortmannin plus GLUT-1 AS-ODN group was lower than that in the control group (p < 0.05, Fig 3). Wortmannin plus GLUT-1 AS-ODN enhanced the effect of inhibition of expression of GLUT-1, p-Akt, and PI3K mRNA significantly versus wortmannin or GLUT-1 AS-ODN alone (p < 0.05, Fig 3).

After X-ray irradiation, wortmannin alone, and wortmannin plus GLUT-1 AS-ODN decreased the expression of GLUT-1, PI3K, and p-Akt mRNA significantly, compared with the 10-Gy X-ray group (p < 0.05, Fig 3). Wortmannin plus GLUT-1 AS-ODN significantly enhanced the effect of wortmannin alone on the inhibition of expression of GLUT-1 and PI3K mRNA after 10-Gy X-ray irradiation (p < 0.05, Fig 3). The effect of wortmannin plus GLUT-1 AS-ODN on p-Akt mRNA expression was not significant compared with that of wortmannin alone after 10-Gy X-ray irradiation (p > 0.05).

### Effects of GLUT-1 AS-ODN, Ly294002, and wortmannin on GLUT-1, p-Akt, and PI3K protein levels in the xenograft

**Fig 4:**
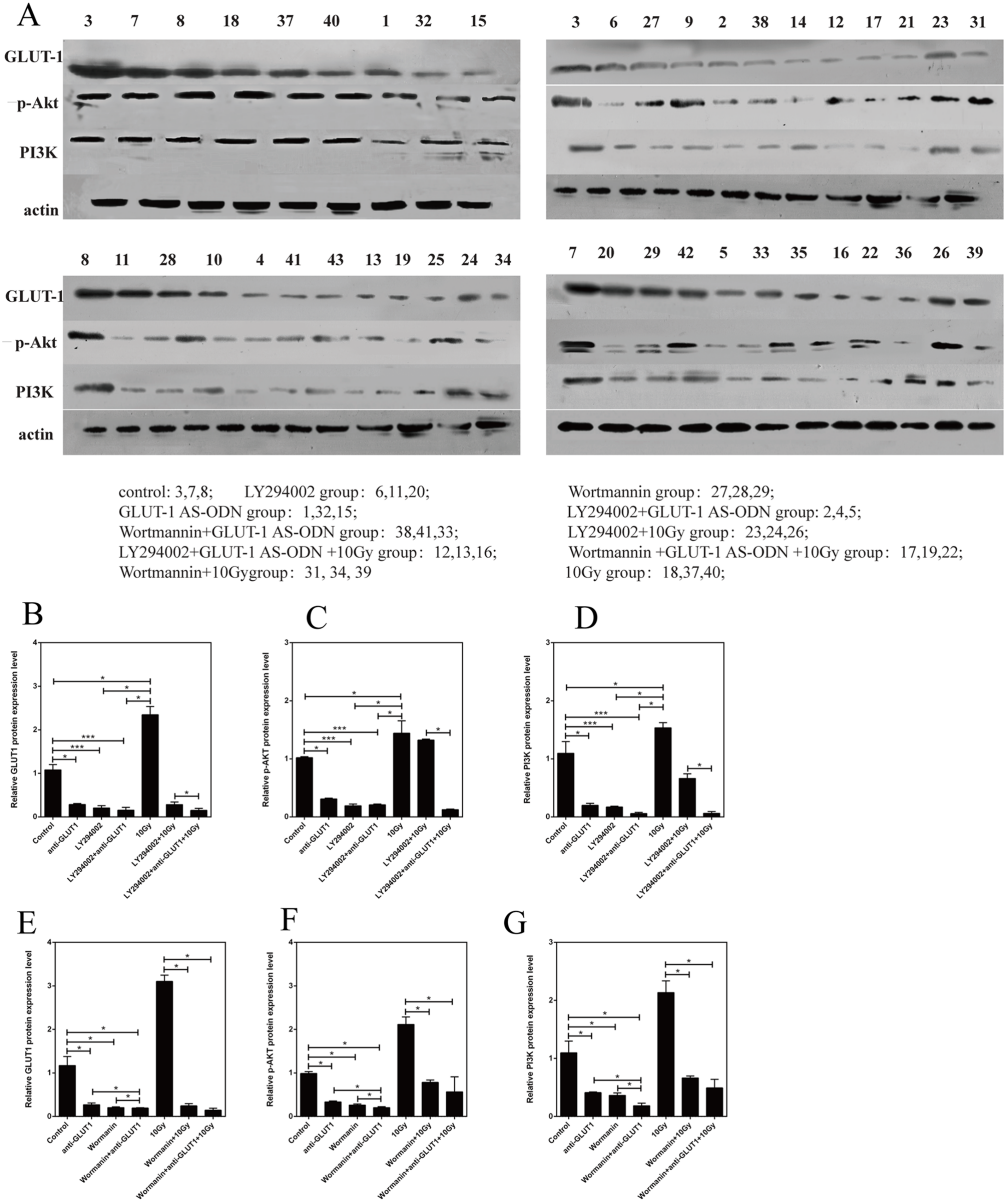
Effects of GLUT-1 AS-ODN, Ly294002, and wortmannin on GLUT-1, p-Akt, and PI3K protein levels in the xenograft. (A) the results of western blotting.(B) The effect of LY294002 on GLUT-1 protein expression. (C) The effect of LY294002 on p-Akt protein expression. (D) The effect of LY294002 on PI3K protein expression. (E) The effect of wortmannin on GLUT-1 protein expression. (F) The effect of wortmannin on p-Akt protein expression. (G) The effect of wortmannin on PI3K protein expression.

Without X-ray irradiation, GLUT-1, p-Akt, and PI3K protein levels after the transfection of GLUT-1 AS-ODN were lower than in the control group (p < 0.05, Fig 4). GLUT-1, p-Akt, and PI3K protein levels in the Ly294002-alone and Ly294002 plus GLUT-1 AS-ODN groups were lower than in the control group, respectively (p < 0.001, Fig 4). However, Ly294002 plus GLUT-1 AS-ODN did not enhance the effects of inhibition of GLUT-1, p-Akt, and PI3K protein levels significantly by Ly294002 or GLUT-1 AS-ODN (p > 0.05, Fig 4).

After X-ray irradiation, the levels of GLUT-1, PI3K, and p-Akt proteins in the 10-Gy X-ray irradiation group were higher than in the control group, but the difference was not significant (p > 0.05). Ly294002 alone and Ly294002 plus GLUT-1 AS-ODN decreased the GLUT-1 and p-Akt protein levels significantly compared with the 10-Gy X-ray group (p < 0.05, Fig 4). Ly294002 plus GLUT-1 AS-ODN enhanced the effect of Ly294002 significantly on the reduction of GLUT-1 and p-Akt protein levels after 10-Gy X-ray irradiation (p < 0.05, Fig 4). However, the effects of Ly294002 alone and Ly294002 plus GLUT-1 AS-ODN on the expression of PI3K protein were not significant (p > 0.05).

Without X-ray irradiation, the GLUT-1, p-Akt, and PI3K protein levels in the wortmannin alone and wortmannin plus GLUT-1 AS-ODN groups were lower than in the control group (p < 0.05, Fig 4). However, wortmannin plus GLUT-1 AS-ODN significantly enhanced the effects of inhibiting the expression of GLUT-1, p-Akt, and PI3K proteins versus wortmannin or GLUT-1 AS-ODN (p < 0.05, Fig 4).

After X-ray irradiation, wortmannin and wortmannin plus GLUT-1 AS-ODN decreased the level of GLUT-1, PI3K, and p-Akt proteins significantly, compared with the 10-Gy X-ray group (p < 0.05, Fig 4). However, this effect of wortmannin plus GLUT-1 AS-ODN in inhibiting GLUT-1, PI3K, and p-Akt protein expression was not significantly different than the effect of wortmannin alone after 10-Gy X-ray irradiation (p > 0.05).

## Discussion

The mechanism of radioresistance in laryngeal carcinoma remains unclear. In a previous study, we found that overexpression of GLUT-1 may be associated with radioresistance of laryngeal carcinoma1. Further studies showed that targeted suppression of GLUT-1 expression by antisense oligodeoxynucleotides (AS-ODN) might enhance the radiosensitivity of laryngeal carcinoma [1]. Thus, we suggest that high expression of GLUT-1 may play an important role in the radioresistance of laryngeal carcinomas. However, this radioresistance is likely the result of the interactions of multiple factors.

Widely used ATP-competitive inhibitors of PI3K in the laboratory setting include wortmannin and LY294002. They have shown anti-proliferative and pro-apoptotic effects in preclinical in vitro and in vivo studies[22]. LY294002 is the first compound to act as a specific inhibitor for the ATP-binding site of PI3K, which can inhibit PI3K activity and Akt activation[25,26]. Wortmannin is a specific inhibitor of the enzymatic activity of the p110 subunit of PI3K, which also can inhibit PI3K activity and Akt activation, but has no inhibition on other intracellular kinases and mitochondrial enzyme[27]. GLUT-1 AS-ODN (antisense oligodeoxynucleotide) can target suppress the expression of GLUT-1 which could enhance the radiosensitivity of laryngeal carcinoma[1]. In the present study, we found that Wortmannin and LY294002 separately combined with radiation had similar effects on tumor volume, tumor weight, rate of tumor growth and apoptosis in tumors..

In a recent study, we found that the effects of apigenin on inhibiting xenograft growth and enhancing xenograft radiosensitivity might be associated with suppressing the expression of GLUT-1 via the PI3K/Akt pathway. In addition, apigenin may enhance the effects of GLUT-1 AS-ODNs via the same mechanism [28]. However, apigenin is not a specific inhibitor of the PI3K/Akt signaling pathway, as we found that the levels of GLUT-1, Akt, and PI3K mRNA were higher in the 50 μg apigenin plus 10 Gy group compared to the 10 Gy group,So there may not have a very good specificity. While in the present study, wortmannin and LY294002 are the specific inhibitors of PI3K, so we can better understand the role of PI3K/Akt pathway in the radiosensitivity of laryngeal carcinoma and its relationship with GLUT-1[28].

In a recent study, we also found that overexpression of GLUT-1 and PI3K/Akt may play a role in the chemoresistance of laryngeal carcinoma Hep-2 cells. Apigenin may enhance the chemoresistance of Hep-2 cells by co-suppression of the expression of GLUT-1 and p-Akt [14]. Thus, we considered that overexpression of GLUT-1 and PI3K/Akt may also play a role in radioresistance in Hep-2 cells, and that radiosensitivity may be enhanced by suppressing the expression of GLUT-1 and the activity of the PI3K/Akt signaling pathway.

In the present study, we found that the xenograft size and weight in the 10-Gy group were not decreased significantly versus the control group. We again showed that 10-Gy X-ray irradiation increased the expression of GLUT-1 mRNA significantly in xenografts of Hep-2 cells. In our previous study, we found that overexpression of GLUT-1 mRNA and protein may be a cause of radioresistance in laryngeal carcinomas, *in vitro* and *in vivo* [1].

Several other studies have reported similar findings. Pedersen et al. found that GLUT-1 expression may be associated with radiation resistance in two sublines from the lung cell lines CPH 54A and CPH 54B[29]. Doki et al. found high expression of GLUT1 in squamous cell carcinoma of the esophagus (ESCC) after radiotherapy[30]. Huang et al. found that GLUT-1 alone or co-expression of CD147 and GLUT-1 showed greater resistance to radiotherapy[31].

Possible mechanisms of radioresistance in cancer caused by overexpression of GLUT-1 were discussed in our previous report [1]. Briefly, first, increased expression of GLUT-1 may meet the higher energy demands of cancer cells. Second, GLUT-1 is an important intrinsic hypoxic marker, and hypoxia may induce radioresistance in cancer cells. Third, CD133+ carcinoma stem cells may be a cause of radioresistance[32], and we found that GLUT-1 expression in CD133+ laryngeal carcinoma was higher than that in CD133- laryngeal carcinoma cells[33].

In the present study, we found that the expression of p-Akt and PI3K mRNA and GLUT-1 protein in the 10-Gy X-ray irradiation group were higher than those in the control group; however, the difference was not significant. These results suggested that an activated PI3K/Akt signal pathway might be involved in the GLUT-1-induced mechanism of resistance or insensitive to radiotherapy in laryngeal carcinoma. Some studies have suggested that the PI3K/Akt pathway may be involved in GLUT-1 trafficking and activity [34,35].

An activated PI3K/Akt signaling pathway may be related to the proliferation of tumor cells, the activation of which is closely related to poor prognosis and resistance to cancer radiotherapy [34,35]. AKT is involved in regulating energy metabolism in cancer cells, enhancing glycolytic regulators, such as GLUT-1 expression[36]. PI3K/Akt/mTOR signaling is involved in several cancer cell metabolic processes, including glycolysis[37]. AKT activation leads to increased GLUT-1 expression and translocation to the membrane, resulting in greater glucose uptake [38]. Some studies have demonstrated that inhibition of the PI3K/Akt signaling pathway may decrease the expression of GLUT-1, causing growth inhibition in cancer cells [18,19,37]. Melstrom et al. found that both apigenin and PI3K/Akt inhibitors may downregulate GLUT-1 expression in human pancreatic cancer cells *in vitro* [18]. Yang et al. found that miR-21 may suppress glycolytic enzymes, including GLUT-1, and suppress high glycolysis levels in bladder cancer cells via inhibition of the PI3K/Akt/mTOR pathway[35]. Rashmi et al. found that AKT inhibitors may inhibit glucose uptake via decreased delivery of GLUT-1 and GLUT-4 to the cell membrane in cervical cancer. They suggested that AKT inhibitors might improve sensitivity to chemoradiation in cervical cancer [37].

However, no data on enhancing radiosensitivity by combined inhibition of PI3K/Akt and GLUT-1 expression or suppression of expression of GLUT-1 via inhibition of the PI3K/Akt signaling pathway in carcinomas have been reported previously. This is the first reported study to demonstrate that co-inhibition of expression of GLUT-1 and the PI3K/Akt signaling pathway may improve the radiosensitivity of laryngeal carcinoma *in vivo*. Without X-ray irradiation, GLUT-1 AS-ODN and wortmannin reduced the size of tumors significantly, compared with the control (p<0.05). Ly294002 or wortmannin combined with GLUT-1 AS-ODN also reduced the size of tumors significantly, compared with the control (p < 0.05). After 10-Gy X-ray irradiation, Ly294002, wortmannin, Ly294002 plus GLUT-1 AS-ODN, and wortmannin plus GLUT-1 AS-ODN reduced the size of tumors significantly versus the 10-Gy group (p < 0.05). The results in terms of weight and apoptosis in the xenografts were similar after the mice were euthanized. GLUT-1 AS-ODN enhanced the rate of tumor growth inhibition and apoptotic rate significantly of Ly294002 or wortmannin after X-ray irradiation. This suggests that GLUT-1 AS-ODN combined with PI3K/Akt inhibitors made the cells more sensitive to X-ray irradiation. Thus, these results indicate that GLUT-1 AS-ODN and PI3K/Akt inhibitors were radiosensitizers (E/O values of Ly294002, wortmannin, and wortmannin plus GLUT-1 AS-ODN were 2.7, 1.8, and 1.8, respectively, all >1.4).

Next, we investigated the molecular mechanisms underlying the enhanced radiosensitivity of laryngeal carcinoma *in vivo*. GLUT-1 AS-ODN, Ly294002, and wortmannin may decrease the levels of GLUT-1, p-Akt, and PI3K mRNA and protein significantly, compared with the control, without irradiation. GLUT-1 AS-ODN enhanced the effect of inhibiting the expression of GLUT-1, p-Akt, and PI3K mRNA by wortmannin and Ly294002 alone. After X-ray irradiation, GLUT-1 AS-ODN, Ly294002, and wortmannin decreased the expression of GLUT-1, p-Akt, and PI3K mRNA significantly compared with the 10-Gy group. GLUT-1 AS-ODN enhanced the effect of inhibiting the expression of GLUT-1, PI3K, and p-Akt mRNA significantly of Ly294002 after 10-Gy X-ray irradiation. Wortmannin alone and wortmannin plus GLUT-1 AS-ODN decreased the levels of GLUT-1, PI3K, and p-Akt proteins significantly compared with the 10-Gy X-ray group. Ly294002 affected GLUT-1 and p-Akt protein levels primarily. These findings are consistent with the effect of GLUT-1 AS ODN and PI3K/Akt inhibitors on the size and weight, rate of tumor growth inhibition, and apoptotic rate. We suggest that co-inhibition of GLUT-1 expression and the activation of the PI3K/Akt signaling pathway may improve the radiosensitivity of laryngeal carcinoma *in vivo*, the mechanism(s) of which may involve inhibiting the expression of GLUT-1, p-Akt, and PI3K via GLUT-1 AS-ODN and PI3K/Akt inhibitors. Moreover, GLUT-1 ASODN may enhance the effects of PI3K/Akt inhibitors on the expression of these markers.

## Conclusions

In the present study, overexpression of GLUT-1 and PI3K/Akt signaling pathway components may be involved in the radioresistance of laryngeal carcinoma *in vivo*. Co-inhibition of GLUT-1 expression and activation of the PI3K/Akt signaling pathway may improve the radiosensitivity of laryngeal carcinoma *in vivo*.
